# Addressing malaria vector control challenges in South Sudan: proposed recommendations

**DOI:** 10.1186/1475-2875-12-59

**Published:** 2013-02-08

**Authors:** Emmanuel Chanda, Constantino Doggale, Harriet Pasquale, Robert Azairwe, Samson Baba, Abraham Mnzava

**Affiliations:** 1 Population Services International, Juba, Republic of South Sudan; 2 Ministry of Health, National Malaria Control Programme, Juba, Republic of South Sudan; 3 Management Sciences for Health, Juba, Republic of South Sudan; 4 Global Malaria Programme, World Health Organization, Geneva, Switzerland

## Abstract

Upon the signing of the Comprehensive Peace Agreement in 2005, the Republic of South Sudan (RSS) has faced a lot of challenges, such as a lack of infrastructure, human resources and an enormous burden of vector borne diseases including malaria. While a national malaria strategic plan 2006-2011 was developed, the vector control component has remained relatively weak. The strategy endorses the distribution of long-lasting insecticidal nets (LLINs) as the frontline intervention with other interventions recommended only when technical and institutional capacity is available. In 2006, a draft integrated vector management (IVM) strategic plan 2007–2012 was developed but never implemented, resulting in minimal coordination, implementation and coverage of malaria vector control tools including their inherent impact. To address this challenge, the vector control team of the National Malaria Control Programme (NMCP) is being strengthened. With the objective of building national capacity and technical collaboration for effective implementation of the IVM strategy, a national malaria vector control conference was held from 15-17^th^ October 2012 in Juba. A range of NMCP partners, state ministries, acadaemia, private sector, national and international non-governmental organizations, including regional and global policymakers attended the meeting. The conference represented a major milestone and made recommendations revolving around the five key elements of the IVM approach. The meeting endorsed that vector control efforts in RSS be augmented with other interventions within the confines of the IVM strategy as a national approach, with strong adherence to its key elements.

## Background

The Republic of South Sudan (RSS) is a heartland of several vector-borne diseases, including malaria [1]. *Anopheles gambiae* s.s*, Anopheles arabiensis* and *Anopheles funestus* s.s are the major vectors of malaria in the country. Upon the signing of the Comprehensive Peace Agreement in 2005, a national malaria strategic plan 2006-2011was developed [2]. One of the key objectives of the strategy was to “*increase the population coverage with effective malaria prevention as part of an integrated vector control strategy that utilizes all approaches, including long-lasting insecticidal nets* (LLINs)*, indoor residual spraying* (IRS) *and larval source management (larviciding and environmental management) when and where most suitable and sustainable”.* However, due to lack of specialized technical capacity, appropriate infrastructure and sustainable financing in the post conflict environment, to date vector control has largely consisted of promoting the use of LLINs distributed through periodic mass campaigns (universal coverage) and via routine Antenatal Care (ANC) and Expanded Programme for Immunization (EPI) services to pregnant women and infants respectively [3].

Since 2008, more than seven million nets have been provided free-of-charge to recipients across the country [3]. However, household ownership, and more importantly, the use of LLINs by vulnerable groups have remained lower than is required to provide vector control benefits of the intervention. The 2009 Malaria Indicator Survey found that 53% of households in South Sudan owned an ITN; 25% of children less than five years of age and 36% of pregnant women had slept under an ITN the night before the survey [4]. The Sudan Household and Health Survey (SHHS) 2010 found a lower household ownership of LLINs at only 34.2 percent [5]. While a more recent survey conducted by NetWorks/Malaria Consortium in Lainya County (April 2012) found higher household ownership of ITNs (66.3%), use of the nets was still low with only 27% of children having used the nets [6].

To strengthen malaria vector control, the Ministry of Health (MoH) has adopted the integrated vector management (IVM) strategy for the control and prevention of vector-borne diseases – including malaria [7]. This followed the endorsement of IVM as a regional strategic approach through a WHO Regional Committee Resolution (EM/RC52.R6) in 2005 for the Eastern Mediterranean [8].

**Figure 1:**
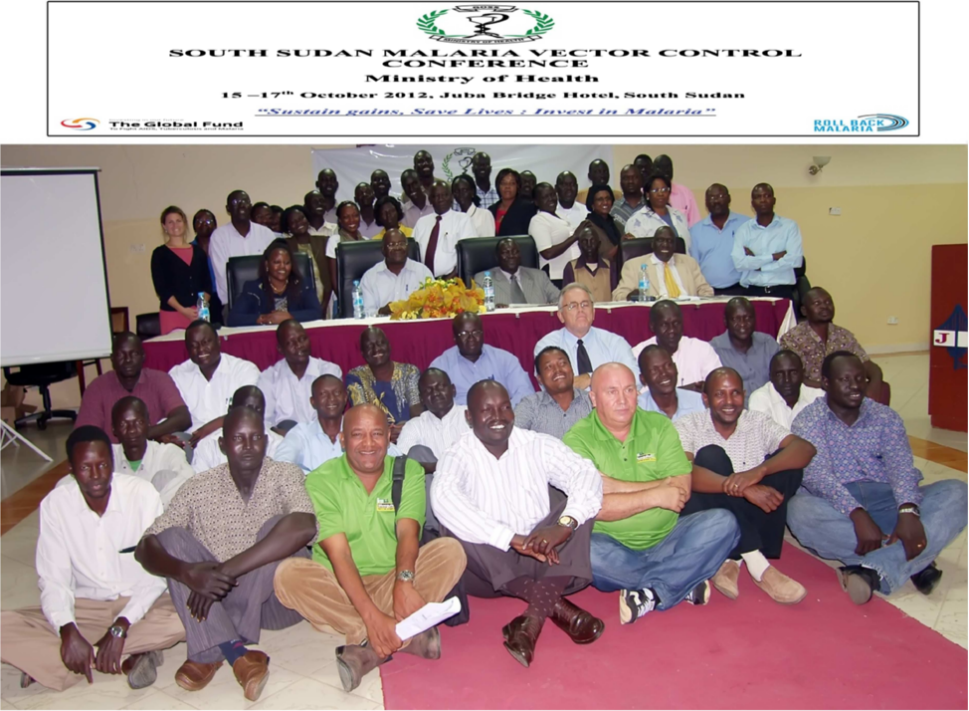
Some of the participants of the South Sudan Malaria Vector Control Conference.

In this regard, the MoH with the support of the Global Fund to fight Tuberculosis, HIV/AIDS and Malaria (GFATM) organized the first ever national malaria vector control conference from 15-17^th^ October 2012 in Juba to; 1) update malaria stakeholders on the IVM Global strategic framework as a WHO recommended approach for vector control; 2) review current LLIN implementation approaches, share experiences and propose means of increasing coverage and use of the intervention; 3) discuss the role and implementation arrangements for other malaria vector control interventions (IRS and LSM) and; 4) review the draft IVM strategic plan and discuss the engagement of other vector-borne disease control programmes. A range of NMCP partners, state ministries, academia, private sector, national and international non-governmental organizations, including regional and global policymakers attended the meeting (Figure 1; Additional file 1).

## WHO-led IVM strategy as main approach for malaria vector control in RSS

IVM is defined as ‘*a rational decision-making process for the optimal use of resources for vector control*’. Its goal is to make a significant contribution to the prevention, reduction or interruption of transmission of vector-borne diseases. The principles and approaches to IVM are set out in the Global Strategic Framework for IVM [9]. Characteristic features of IVM include: methods based on knowledge of factors influencing local vector biology, disease transmission and morbidity; use of a range of interventions, often in combination and synergistically; collaboration within the health sector and with other public and private sectors that impact on vectors; engagement with local communities and other stakeholders; a public health regulatory and legislative framework. The IVM strategy has five key elements, these are: advocacy, social mobilization and legislation; collaboration within the health sector and with other sectors; integrated approach; evidence-based decision-making; and capacity-building.

In the RSS, the WHO-led IVM has been adopted as the main platform for vector control. A national vector control needs assessment has been conducted to thoroughly analyse the country requirements for a proper implementation of IVM through the determination of existing gaps in policies, strategies, legislation and capacity for improving vector control in view of IVM and its integration in the existing framework of national health policies in the RSS [10]. A draft strategic plan for IVM has been developed for the period 2007–2012.

## Review of LLIN deployment, gaps and opportunities for increasing their use in RSS

While deployment of LLINs remains the frontline intervention for malaria vector control, distribution and utilization has consistently been below the set targets necessitating the need to review the LLIN guidelines, policies and distribution strategies with a view of improving coverage and use. Effective vector control aims to deploy interventions before the peak transmission season. However, there are notable gaps and challenges of coordination and communication, weak intersectoral collaboration at all levels, limited information on retention and use of LLINs, preferences by communities, vector bionomics and their insecticide resistance profiles, environmental and human safe guards. WHO recommends mass LLIN distribution conducted just before the transmission period targeting an average of about one net for every 1.8 people [11]. Community derived figures could be useful in quantifying the needed nets to maximize distribution that should be confined to the short window of dry season for logistical ease.

It is critical to strengthen routine distribution and explore the potential of other avenues for LLINs distribution such as community-based LLIN continuous distribution, including other replacement mechanisms to tackle the challenges of low LLIN durability - mainly physical integrity due to wear and tear and to a lesser extent due to insecticide residual efficacy [12]. Improved awareness campaign to increase involvement of churches, schools, the private sector and political will, including research into establishing ownership and correct net use and entomological baseline data collection is equally necessary.

## Role and implementation arrangements for other vector control interventions in RSS

Given the unique context of South Sudan in having a long peak malaria transmission season (lasting 7–8 months in the southern parts and 5–6 months in the northern regions) and infrastructural challenges, deployment of LLINs remains the frontline intervention for malaria vector control [2]. However, the epidemiological landscape of malaria necessitates the deployment of a multi-pronged approach including painstaking implementation of IRS guided by local data on insecticide resistance in malaria vectors [13], while slowly introducing LSM as a supplementary measure for malaria control with strong community involvement. While RSS has for a long time been viewed not to be amenable for deployment of IRS, the last six years has seen unprecedented infrastructure development including housing structures that are now ideal for spraying. Following interim recommendations by WHO on larviciding [14], the intervention is unlikely to be sustained for most rural areas of the RSS. The intervention, particularly larviciding with *Bacillus thuringensis* var. *israelensis* or insect growth regulators, remains recommended for the urban areas of the country where breeding sites are few, fixed and findable. Effective control of malaria vectors will require exploring new innovations of mosquito control technologies, including biological control methods, such as the use of larvivorous fish *Gambusia affinis* in targeted areas.

Generally improved vector control entails capacity building which is not only the responsibility of public institutions but also private institutions [9]. Currently there are 19 people who have received MSc level training in Medical Entomology and Vector Control in South Sudan. These capacities could better be harnessed for the implementation of the IVM strategy. Equally, establishment of monitoring sentinel sites for tracking vector bionomics including their insecticide resistance profiles presents an opportunity for guiding deployment of pilot IRS in at least three state capitals in South Sudan. This will safeguard the limited classes of insecticides through a rational resistance management strategy from the outset [13].

## Reviewing the draft IVM strategic plan and engaging other vector borne disease control programmes in RSS

The MoH/NMCP needs to take a leading role in malaria control and hold a stakeholders consensus workshop to discuss the modalities of engaging other vector-borne diseases and IVM consolidation through building of core capacities and implementation of new interventions. Incorporation of other vector-borne diseases requires establishment of a multi-sectoral steering committee to update the IVM strategic plan, to develop the technical guidelines, and spearhead their implementation. The current draft IVM strategic plan requires systematic reviewing with a wider representation of participants from other vector borne disease control programmes [7]. While integration of other vector borne diseases has been shown to open a window for leveraging resources, government needs to advocate for more funding for both implementation of interventions and capacity building. Strengthening entomological monitoring, particularly vector bionomics and their insecticide resistance profiles will optimize resource utilization in areas of co-endemicity of vector borne diseases. The expansion phase should involve the future scaling-up of IVM interventions in all possible states and counties in the country [10].

## Learning from the experience of other countries

Through the IVM strategy, some countries have put malaria vector control high on the political agenda and are making steady progress towards focalized elimination of the disease [11]. In Zambia and Eritrea, key interventions, IRS and LLINs, are deployed as main thrust intervention with larviciding and environmental management as supplementary tools in accordance with and strict adherence to a set of eligibility criteria [15,16]. Educating communities and empowering local authorities to prevent net abuse, conducting door to door visits to ensure immediate proper hanging and use of the nets has enhanced effectiveness of nets [6]. There is need to have a strong entomological team at national level to coordinate routine monitoring of resistance and data analysis and interpretation to inform policy decisions, translate policies and guidance into action at ground level [13]. This will also need well trained entomologists at state level to help in monitoring, etc. In the short term, the NMCP need to establish external linkages with international research institutions to build further entomological capacity. Strengthening environmental safeguards through collaboration with in-country environmental regulatory bodies is also necessary.

## Recommendations

Although RSS is a post conflict environment characterized by a multiplicity of challenges, the potential to implement effective malaria vector control is enormous. The successful conference on strengthening malaria vector control within the IVM framework by reviewing the available and ongoing strategies and identifying complementary interventions represent a major milestone. The key recommendations of the conference revolving around the five key elements of the IVM approach were:

1. The Republic of South Sudan (RSS) need to put malaria vector control on the political agenda of the government to advocate for national and international vector control resources in order to sustain the effort.

2. Vector control is currently being implemented almost entirely by international NGOs, LLINs (Population Service International, World Vision, IMA and Merlin) and pilot IRS (Mentor Initiative). As national institutional capacity-including those of the communities is strengthened, it is necessary now to empower the NMCP to manage and coordinate the implementation of malaria vector control.

3. To ensure that LLINs universal coverage is sustained and utilization improved, mass campaign should be conducted every two years based on the population figures generated from the local communities with strengthened routine distribution via ANC and EPI as well as continuous community distribution and IEC/BCC.

4. Develop implementation frameworks for IRS including national insecticide policy and legislation, private sector involvement and guidelines for deploying pilot IRS programmes in state capitals because of logistical challenges in rural areas.

5. Recognizing the recent recommendations by WHO, Larval Source Management using larviciding with WHOPES recommended products will only be amenable as a supplementary pilot intervention in selected urban areas where breeding sites are few, fixed and findable.

6. To guide the choices and overall implementation of vector control interventions, there is need to establish sentinel sites across the country for entomological (vector bionomics and insecticide resistance) monitoring- including data on LLIN life span in the field, along with the establishment of a national group of experts to analyse and interpret resistance data to guide national policies and procurement decisions.

7. To be able to measure the impact of vector control implementation, it would require strengthening epidemiological monitoring through a robust malaria case surveillance system and HMIS to improve malaria morbidity and mortality data.

8. To ensure high quality of vector control commodities and equipment, decisions on their procurement should be evidence-based and compliant with the legally binding tender documents that follow the WHO guidelines on procurement [17].

9. Establish national inter-sectoral committee to update the IVM strategy, integrate other vector-borne diseases and oversee planning and implementation of IVM at central, state and county levels.

10. Strongly advocate for Government support and commitment in strengthening human resource and institutional capabilities for vector control at state and county levels.

## Conclusions

The main endorsement of the meeting was that current vector control efforts in RSS be augmented with other interventions within the confines of the IVM strategy as a national approach, with strong adherence to the five key elements of the approach. Implementation of these recommendations must be a shared responsibility of all partners and other relevant sectors of government (agriculture, housing, environment, local government, finance, education). As a first step, establishing a functional national intersectoral coordination mechanism as part of IVM, will create a platform in which these recommendations can be implemented cost-effectively. This would also require the updating of the IVM strategic plan as a key component of the overarching National Malaria Control Strategy.

## Abbreviations

ANC: Ante-Natal Clinic; BCC: Behavioural Change Communication; EPI: Expanded Programme for Immunization; GFATM: Global Fund to HIV/AIDS, Tuberculosis and Malaria; HMIS: Health Management Information System; IEC: Information, Education and Communication; IRS: Indoor Residual Spraying; IVM: Integrated Vector Management; LLIN: Long-Lasting Insecticidal Nets; LSM: Larval Source Management; MoH: Ministry of Health; NGO: Non Governmental Organization; NMCP: National Malaria Control Programme; RSS: Republic of South Sudan; SHHS: Sudan Household and Health Survey; WHO: World Health Organization; WHOPES: World Health Organization Pesticide and Evaluation Scheme.

## Competing interests

The authors declare that they have no conflict of interest.

## Authors’ contributions

EC conceived the idea and drafted the manuscript, and CD, HP, RA, SB, and AM collaborated and critically reviewed the article. All authors read and approved the final manuscript.

## Supplementary Material

Additional file 1 Conference Agenda. Click here for file
