# Supplementary material for: Addressing malaria vector control challenges in South Sudan: proposed recommendations
Source: Malar J. 2013 Feb 8;12:59. doi: 10.1186/1475-2875-12-59 (PMC3570318; doi:10.1186/1475-2875-12-59)
Supplement: Additional file 1 — Conference Agenda. [file 1475-2875-12-59-S1.docx]

**Additional file 1: Conference Agenda**

| **CONFERENCE Agenda** | | |
| --- | --- | --- |
| **Sunday October 14, 2012** | | |
| **Time** | **Activity** | **Facilitator / Presenter** |
| 1200 - 2200 | Arrival of Participants | Secretariat |
| **Day One, Monday October 15, 2012** | | |
| **Time** | **Activity** | **Facilitator / Presenter** |
| 0900 | Registration | Secretariat |
| 0920 | Introductions and Administrative arrangements | Secretariat |
| 0930 | Expectations, Fears, Ground Rules of the meeting | Chairperson |
| 0945 | Conference Goals, Objectives and Outputs | Chairperson |
| 1000 | Official opening remarks | The Guest of Honour |
| **Objective 1** | *To update malaria stakeholders on the Integrated Vector Management (IVM) Global strategic framework as a WHO recommended approach for vector control;* | |
| 10:15 | An overview of malaria control in South Sudan | Dr. Harriet Pasquale |
| 10:45 | ***Tea Break*** | All |
| 11:00 | Biological attributes of malaria vectors: essentials for control | Dr. Emmanuel Chanda |
| 11:30 | Addressing malaria vector control challenges and threats in the framework of IVM – an update | Dr. Abraham Mnzava |
| 12:30 | Plenary discussions | All |
| 1300 | *Lunch Break* | All |
| **Objective 2** | ***To review current LLIN/ITN implementation approaches, share experiences and propose means of increasing coverage and use of the intervention;*** | |
| 1400 | Insecticide Treated Nets guidelines and distribution strategies in South Sudan | Dr. Emmanuel Chanda |
| 1440 | Experiences of LLINs mass campaigns in South Sudan | PSI |
| 1520 | Community-based LLIN continuous distribution Pilot Project | Malaria Consortium |
| 1600 | Plenary discussions | All |
| 1630 | *Tea Break/Day Ends* | All |
| **Day Two, Tuesday October 16, 2012** | | |
| **Time** | **Activity** | **Facilitator / Presenter** |
| 0900 | Summary and Key Points of Day One | Dr. Robert Azairwe |
| **Objective 2** | ***To review current LLIN/ITN implementation approaches, share experiences and propose means of increasing coverage and use of the intervention*** | |
| 0915 | State experience in Western Bahr el Ghazal | Western Bahr el Ghazal |
| 0955 | State experiences in Western Equatoria | Western Equatoria |
| 1035 | Plenary discussion | All |
| 1045 | ***Tea Break*** | All |
| 1100 | Guided discussion on current LLIN/ITN approach | Dr. Robert Azairwe |
| 1300 | ***Lunch Break*** | All |
| **Objective 3** | ***Discuss the role of, and implementation arrangements for other malaria vector control interventions (IRS) in South Sudan*** | |
| 1400 | Indoor Residual Spraying: Steps Towards Implementation | Dr. Emmanuel Chanda |
| 1500 | Country experience: Ethiopia | NMCP (Ethiopia) |
| 1530 | Country experience: Zimbabwe | Reagent Laboratories |
| 1600 | Country experience: Zambia | Dr. Emmanuel Chanda |
| 1610 | ***Tea Break/Day Ends*** | All |
| **Day Three, Wednesday October 17, 2012** | | |
| **Time** | **Activity** | **Facilitator / Presenter** |
| 0900 | Summary and Key Points from Previous Day | Dr. Robert Azairwe |
| **Objective 4** | *Discuss the role of, and implementation arrangements for other malaria vector control interventions (LSM) in South Sudan;* | |
| 0915 | Larval source management for malaria vector control: A focus on larviciding | Dr. Abraham Mnzava |
| 1015 | Private sector Experiences in LSM | Reagent Laboratories |
| 1045 | ***Tea Break*** | All |
| 1100 | Private sector Experiences in LSM | Green Tech MosquitoNix |
| 1130 | Insecticide Resistance Monitoring | Dr. Emmanuel Chanda |
| 1230 | Plenary discussion | All |
| 1300 | ***Lunch Break*** | All |
| **Objective 5** | *To review the current IVM strategic plan for South Sudan and discussing the engagement of other vector borne disease control programmes* | |
| 1400 | IVM steps towards Implementation | Dr. Emmanuel Chanda |
| 1500 | Current IVM strategic plan | Mr. Constantino Doggale |
| 1530 | Plenary discussion | All |
| 1540 | *Recommendations /Next steps* | Secretariat |
| 1600 | *Official Closing Remarks* | The Guest of Honour |
|  | **END OF WORKSHOP** |  |
| 1630 | Secretariat Meeting | Dr. Harriet Pasquale |
| **Thursday, 18^th^ October, 2012** | | |
| **Time** | **Activity** | **Facilitator** |
|  | Departure of Participants | Secretariat |
